# Control of the dual emission from a thermally activated delayed fluorescence emitter containing phenothiazine units in organic light-emitting diodes†

**DOI:** 10.1039/c8ra10393c

**Published:** 2019-02-04

**Authors:** Ikbal Marghad, Fatima Bencheikh, Chao Wang, Sophia Manolikakes, Alice Rérat, Corinne Gosmini, Dae hyeon Kim, Jean-Charles Ribierre, Chihaya Adachi

**Affiliations:** Center for Organic Photonics and Electronics Research (OPERA), Kyushu University Motooka 744, Nishi Fukuoka 819-0395 Japan ribierre@opera.kyushu-u.ac.jp adachi@opera.kyushu-u.ac.jp; Laboratoire de chimie moléculaire LCM, Ecole Polytechnique, CNRS 91128 Palaiseau Cedex France; Japan Science and Technology Agency (JST), ERATO, Adachi Molecular Exciton Engineering Project Fukuoka 819-0395 Japan; Department of Polymer Science and Engineering, Zhejiang University Hangzhou 310027 China

## Abstract

The development of single-component organic dual light-emitting molecules is of interest for a range of applications including white organic light-emitting diodes. Herein, a new thermally-activated delayed fluorescent molecule containing 4,6-bis-phenyl phenothiazine as donor units and 2-thiophene-1,3,5-triazine as acceptor unit was synthesized using a simple cost-effective method. This compound shows two stable molecular conformations due to the presence of the phenothiazine units in its molecular structure. These conformers exhibit different photophysical properties in both solution and thin films. The electroluminescence properties of this novel emitter were then examined in organic light-emitting diodes and the results provide useful insights into the influence of the device architecture on the dual emission characteristics. The experimental results were consistent with the optical simulations and the optimized architecture led to the fabrication of electroluminescent devices with an external quantum efficiency of 11.5% and a maximum luminance value of 10 370 cd m^−2^.

## Abstract 2

A novel TADF emitter showing dual emission is synthesized and applied to OLEDs with an external quantum efficiency of 11.5%.

## Graphical abstract

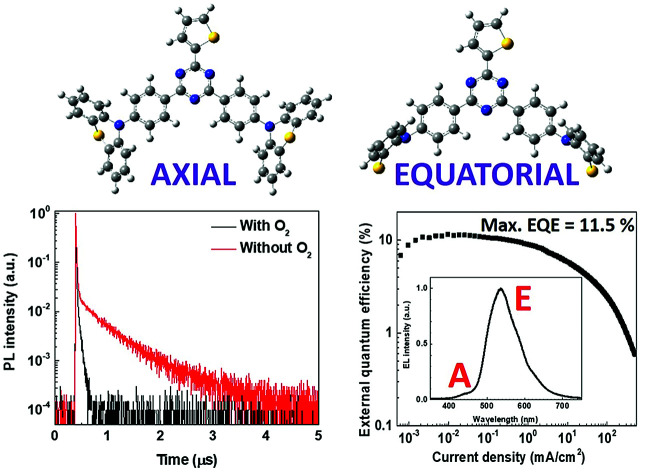

## Introduction

Organic light-emitting diodes (OLEDs) are now used in commercially available display applications and are extremely promising for solid-state lighting sources. Since the demonstration of fluorescent OLED in 1987 by Tang and VanSlyke,^1^ great enhancement of the electroluminescence (EL) quantum efficiency has been achieved using phosphorescence,^2–4^ triplet fusion^5,6^ and thermally-activated delayed fluorescence (TADF).^7–15^ In particular, light-emitting molecules with TADF properties are considered now as the third generation of OLED materials and enable the harvesting of light from both singlet and triplet excitons without the use of any noble heavy metals. A large number of studies has already reported high-performance OLEDs using blue, green, red and near infrared TADF emissive dopants.^16–20^ In most cases, to achieve such a high-efficiency TADF emission, a small singlet–triplet energy gap (Δ*E*_ST_) is crucial to facilitate the upconversion from triplet to singlet excited states *via* thermally-activated reverse intersystem crossing (RISC) process. The most popular approach that have been used to develop TADF OLED emitters showing simultaneously small (Δ*E*_ST_) and increased oscillator strength is based on twisted molecular donor–acceptor architectures with large dihedral angles between the electron-donating and accepting units, resulting in a small spatial overlap between the highest occupied molecular orbital (HOMO) and the lowest unoccupied molecular orbital (LUMO) frontier orbitals.^21–25^ Interestingly, among the large variety of TADF OLED emitters, some of them have been found to exhibit dual emission due to the existence of stable conformers.^26–33^ This class of TADF emitters is evidently of strong interest for white TADF OLEDs and could be promising for other imaging or sensing applications benefiting from a broad emission. Noticeably, while the first example of single-component dual TADF emission was obtained using dyes containing phenothiazine units,^28^ other moieties such as 9,9-dimethyl-9,10-dihydroacridine and phenoxazine have also been recently incorporated into light-emitting dyes with dual stable conformations leading to dual TADF emission during excitations.^26,34^ In this context, the development of innovative TADF molecular architectures with dual conformations for highly efficient OLEDs and a deeper understanding of this dual emission phenomenon are still required.

**Fig. 1:**
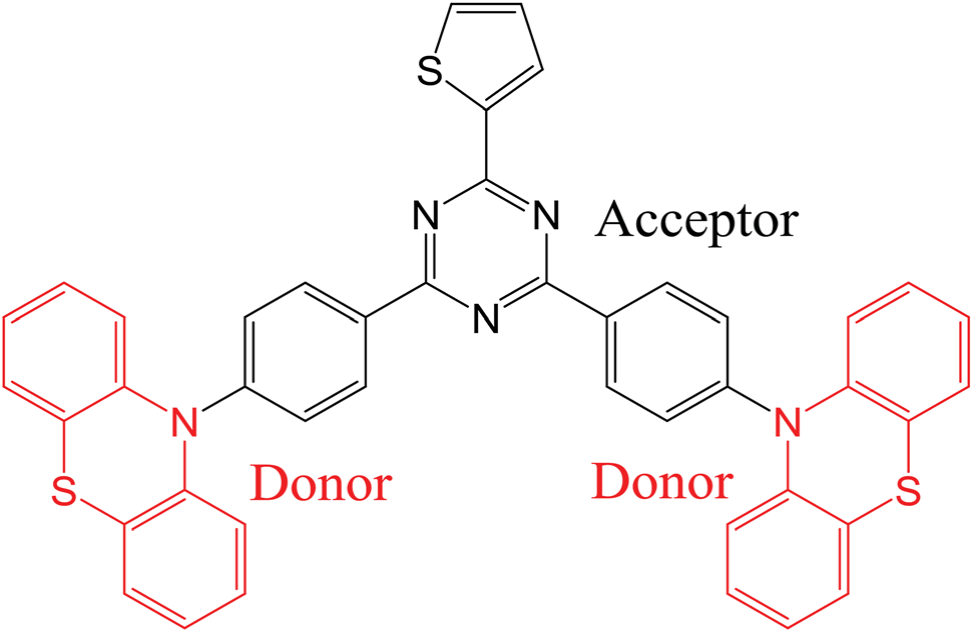
Chemical structure of (T-TRZ)-PTZ.

In this study, we report on the synthesis of a novel TADF emitter ((T-TRZ)-PTZ) containing peripheral phenothiazine donor units and a central 2-thiophene-1,3,5-triazine acceptor unit using a cost-effective synthetic method. A schematic representation of the chemical structure of this dye is shown in Fig. 1. The 2-thiophene unit was herein chosen to shift the dual TADF emission toward longer wavelengths compared to the previously-reported phenyl analogue^29^ and to determine its potential influence on the electronic structure of the molecule.^35,36^ In this context, quantum chemistry calculations as well as the characterization of the photophysical and EL properties of this novel compound are expected to provide insights into the dual emission of TADF molecules containing phenothiazine units. In addition, optical simulations of the EL device properties are carried out to demonstrate the role played by the OLED architecture on the dual emission properties.

## Results and discussion

### Synthesis of (T-TRZ)-PTZ

**Scheme 1:**
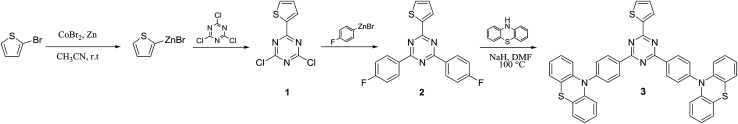
Synthesis of (T-TRZ)-PTZ.

Most of the reaction steps for the synthesis of emitters based on 1,3,5-triazine use expensive catalysts such as palladium. Recently, we reported for the first time a cost-effective and simple synthetic method for the synthesis of TADF emitters incorporating 1,3,5-triazine moieties, which is based on the cobalt catalysed cross coupling of arylzinc with aryl halide.^29^ The important benefit from this approach is related to its production cost which is lower than using palladium catalyst. In the present work, we used this cobalt catalysed cross coupling method for the synthesis of (T-TRZ)-PTZ 3. The synthetic route is based on three main steps as described in Scheme 1. In first place, the mono-substitution reaction of the 1,3,5-triazine with the thiophen-2-ylzinc(ii) bromide catalysed by the cobalt cross-coupling was performed leading to the mono-thiophene triazine intermediate 1 containing two chloro functions that can be further substituted. This first step is then followed by another cobalt cross-coupling between the triazine intermediate 1 and the 4-fluorophenylzinc(ii) bromide, previously prepared from commercially available 4-bromofluorobenzene. This last coupling leads to the triazine intermediate 2 presenting two fluoro functions. The final product 3 was then obtained from 2 by a simple nucleophilic aromatic substitution of phenothiazine in presence of a strong base such as sodium hydride. Detailed chemical characterization of the intermediates and final product, including NMR and HRMS, is provided in ESI (Fig. S1–S4†).

### Quantum chemistry calculations

**Fig. 2:**
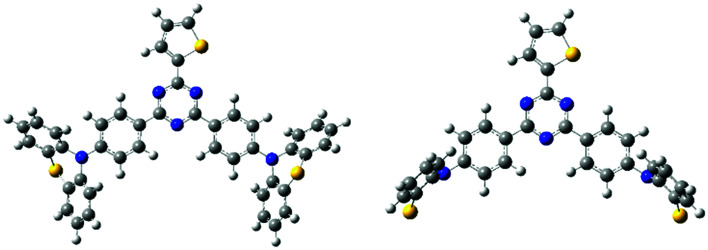
Molecular geometries of the conformers A (left) and E (right) in their ground-state optimized at the B3LYP/6-31G(d) level of theory. Note that only half of the phenothiazine units can be seen in this representation of the conformer E.

**Fig. 3:**
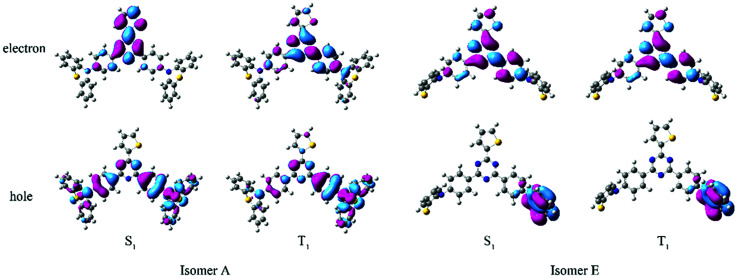
Distribution of the hole and electron NTOs of S_1_ and T_1_ calculated at the B3LYP/6-31G(d) level of theory based on the S_0_ geometry for the conformers A and E. Note that only half of the phenothiazine units can be seen in the representation of the conformers E.

All the quantum chemistry calculations were performed using Gaussian 09.^37^ The electronic structures of (T-TRZ)-PTZ were examined using density functional theory (DFT) and time-dependent density functional theory (TDDFT) with the Tamm–Dancoff approximation (TDA)^38^ for avoiding triplet instabilities.^39^ The ground-state geometries of (T-TRZ)-PTZ were optimized at the B3LYP/6-31G* level. Because of the presence of two phenothiazine units in ((T-TRZ)-PTZ), different stable conformations can be expected for this molecule. As shown in Fig. 2 and Table S1,† one axial (conformer A) and one equatorial (conformer E) configurations are evidenced by the calculations and are found to show minor energy differences (Table S2†). Fig. 3 shows the distribution of the hole and electron natural transition orbitals (NTOs) of S_1_ and T_1_ calculated at the B3LYP/6-31G(d) level of theory based on the S_0_ geometry for both conformers A and E using Multiwfn 3.4.^40^

For each conformer, it is interesting to note that the donor moieties dominate the hole NTOs while the acceptor moieties determine the electron NTOs. Compared to conformer A, the almost identical hole and electron NTOs for the S_0_ → S_1_ and S_0_ → T_1_ charge transfer (CT) transitions in conformer E suggest a vanishing Δ*E*_ST_.^41^ Thus a more facile exciton transformation channel *via* ISC and RISC may process between S_1_ and T_1_. As shown in Table S3,† the two conformers are expected to exhibit different photophysical properties. For instance, calculations indicate that the conformer A should exhibit a strong absorption peak at about 378 nm (3.28 eV) while conformer E should display a flatter and broad absorption band around 532 nm (2.33 eV). The theoretical results also confirm that (T-TRZ)-PTZ should exhibit a dual emission as a direct consequence of the presence of two phenothiazine units in its molecular structure. Importantly, the theoretical results show that the calculated Δ*E*_ST_ values of the conformers A and E in their optimized ground-state geometries are 0.46 eV and 0.01 eV, respectively. This suggests that TADF activity should be significantly more effective in the conformer E. Interestingly, these Δ*E*_ST_ values are slightly different from those calculated in the previously-reported phenyl analogue^29^ (0.0079 and 0.71 eV for the E and A conformers, respectively).

### Photophysical properties of (T-TRZ)-PTZ

**Fig. 4:**
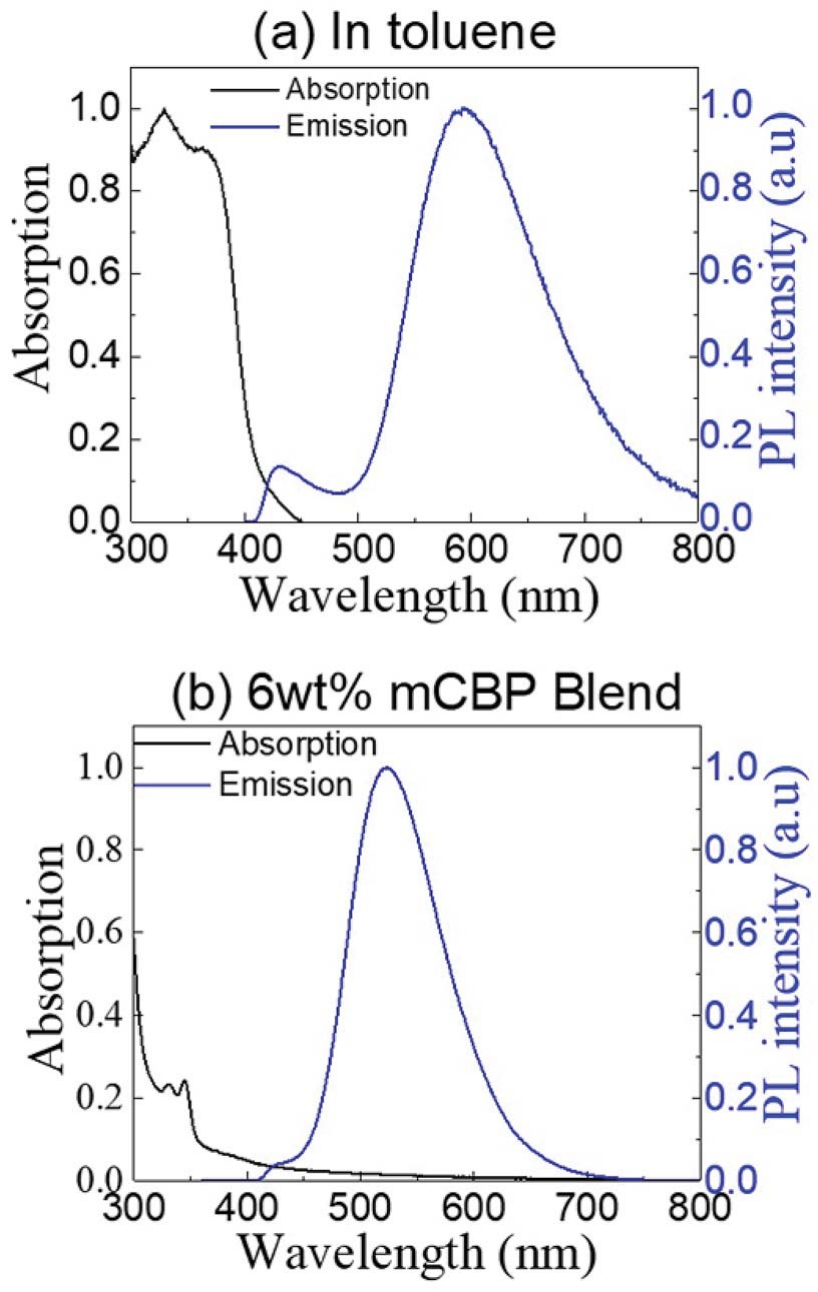
Normalized absorption (black line) and steady-state photoluminescence (blue line) spectra of (T-TRZ)-PTZ (a) in degassed toluene solution with a concentration of 1 × 10^−5^ M and (b) in 6 wt% mCBP blend. The emission spectra were obtained using an excitation wavelength of 340 nm.

The absorption and steady-state photoluminescence (PL) spectra of (T-TRZ)-PTZ measured at room temperature in degassed toluene solution are displayed in Fig. 4a. Concentration of the solution was 1 × 10^−5^ M. The dye (T-TRZ)-PTZ exhibits two main intense absorption bands at 330 and 364 nm, which are assigned to the _1_(π,π*) ← S_0_ transition of the 1,3,5 triazine. In addition, the weak absorption tail at wavelengths longer than 400 nm is related to the intramolecular CT character of the quasi-equatorial conformer of the molecule. The steady-state PL spectrum of (T-TRZ)-PTZ in toluene shows a dual emission with two peaks at around 430 and 593 nm, respectively. The peak at 593 nm shows the highest intensity, a large Stokes shift and a full-width-at-half-maximum of 96 nm, which can be assigned to the emission from the singlet excited state of the quasi-equatorial conformation. The peak at 430 nm presents a significantly lower PL intensity and is attributed to the emission from the singlet excited state of the quasi-axial conformer. Noticeably, the PL emission of (T-TRZ)-PTZ in toluene is about 18 nm red-shifted compared to that of the previously-reported phenyl analogue.^29^ The results indicate that the incorporation of the thiophene unit in (T-TRZ)-PTZ affects the singlet–triplet gap as evidenced by the quantum chemistry calculations and the steady-state PL emission of the molecule.

Fig. 4b shows the absorption and steady-state PL spectra of (T-TRZ)-PTZ doping at 6 wt% a 3,3′-bis(*N*-carbazolyl)-1,1′-biphenyl (mCBP) host. The film was thermally evaporated on top of a fused silica substrate. The mCBP host was selected due to its high triplet energy T_1_, which prevents back energy transfer from the guest to the host molecules and confines the triplets within the guest emitters. Because of the small doping concentration of the guest molecules, the absorption spectrum of the mCBP blend is strongly dominated by the absorption of the host molecules. In particular, the absorption bands observed at 330 and 344 nm are due to the presence of carbazole units in the mCBP molecule.^42^ Noticeably, considering the excitation wavelength of 340 nm, the steady-state PL spectrum of the mCBP blend does not show any emission from the host. This implies that an efficient energy transfer takes place from the mCBP host to the (T-TRZ)-PTZ doping molecules.^43–45^ It can also be seen that the steady-state PL spectrum of the blend film exhibits a dual emission with two peaks located around 423 and 523 nm, respectively. Similarly to the solution case, the low and high energy peaks are attributed to the quasi-equatorial and quasi-axial conformers, respectively. It is worth noting that the emission peak of the quasi-equatorial conformer is significantly blue-shifted in the blend, as compared to its PL spectrum in toluene. This is presumably due to the ICT character of (T-TRZ)-PTZ and the different polarity of the environment.^11,20,46,47^

**Fig. 5:**
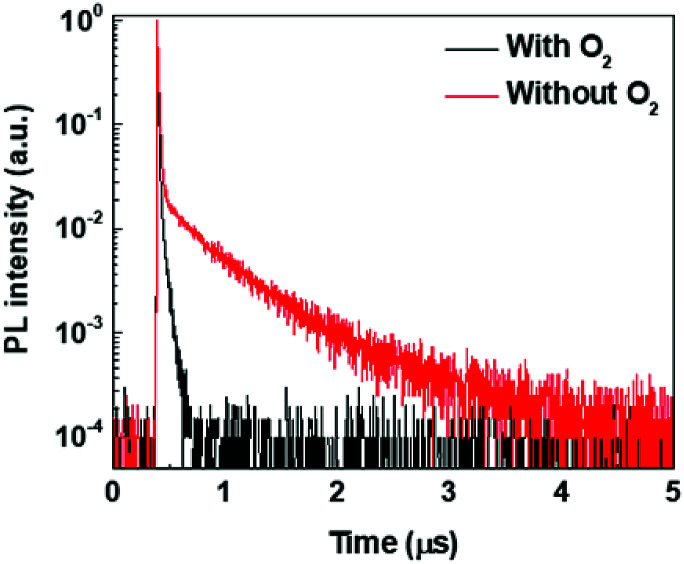
Transient fluorescence decays of (T-TRZ)-PTZ in degased and non-degased toluene solutions. Excitation wavelength was 337 nm and emission wavelength was 593 nm.

To gain further insights into the photophysics of (T-TRZ)-PTZ, the transient PL decay of the equatorial conformer was measured in degased and non-degased toluene solution at a low concentration around 10^−6^ M. Excitation wavelength was 337 nm and the emission wavelength was 593 nm. The kinetics shown in Fig. 5 can be described by the sum of two exponential decay functions. We define the slow and fast time constants characteristics of these exponential decay functions to the delayed and prompt fluorescence lifetimes, respectively. While the prompt fluorescence lifetime does not change with the presence or not of oxygen and is in the order of 10 ns, the delayed fluorescence lifetime is found to increase from 50 ns to 570 ns after degasing the toluene solution. These data indicate that the equatorial conformer of (T-TRZ)-PTZ shows some TADF properties, in accordance with the theoretical results obtained by quantum chemistry calculations. The observed behavior in presence of oxygen shows indeed that the triplet states involved in the TADF process are readily quenched by the oxygen triplet ground state and this fully supports the assignment of TADF. An important parameter which needs to be measured in order to evaluate the potential of (T-TRZ)-PTZ for OLEDs is its photoluminescence quantum yield (PLQY) in thin film. We found that the PLQY of the 6 wt% mCBP blend in nitrogen atmosphere yields a value of 43%.

### Characterization of the OLEDs

**Fig. 6:**
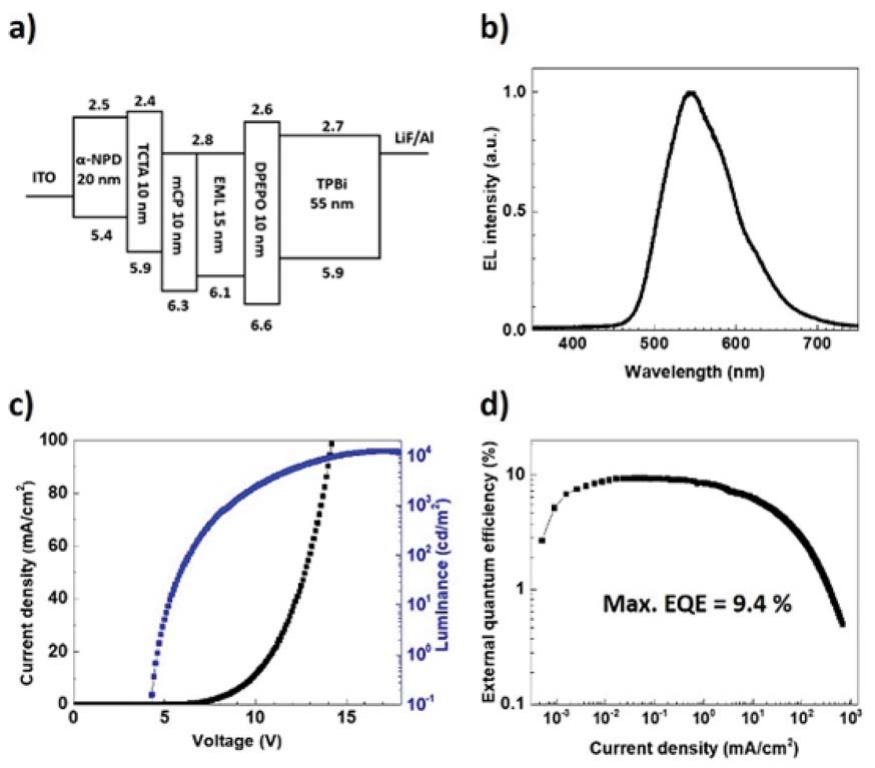
(a) Schematic representation and energy diagram of the OLED structure. (b) Electroluminescence spectra at different current densities. (c) Current density–voltage-luminance characteristic and (d) external quantum efficiency as a function of the current density.

To examine the EL properties of (T-TRZ)-PTZ, the first batch of OLEDs had the following architecture: indium tin oxide (ITO) (100 nm)/*N*,*N*′-diphenyl-*N*,*N*′-bis(1-naphthyl)-1,10-biphenyl-4,4′-diamine (α-NPD) (20 nm)/tris(4-carbazoyl-9-ylphenyl)amine (TCTA) (10 nm)/1,3-bis(*N*-carbazoyl)benzene (mCP) (10 nm)/6 wt% (T-TRZ)-PTZ: mCBP (15 nm)/bis[2-(diphenylphosphino)phenyl]ether oxide (DPEPO) (10 nm)/1,3,5-tris(2-*N*-phenylbenzimidazolyl)benzene (TPBi) (55 nm)/lithium fluoride (LiF) (0.8 nm)/aluminium (Al) (900 nm). A schematic representation and energy diagram of the device structure are provided in Fig. 6a. α-NPD and mCP were chosen for the hole transport layers while DPEPO and TPBi were selected for the hole blocking and electron transport layers, respectively. Fig. 6b–d show the EL characteristics of this OLED. The device exhibits green-yellowish emission with a turn-on voltage of 4.6 V, a maximum current efficiency of 35.47 cd A^−1^, a power efficiency of 11.1 lm W^−1^, a maximum luminance up to 10 370 cd m^−2^ and a maximum EQE of 9.4% without any light outcoupling enhancement. These data could be reproduced in several devices. The obtained EQE value is significantly higher than the upper theoretical limit stated for conventional fluorescent OLEDs. We attribute this result to an efficient TADF process enabling the effective upconversion of the triplets to the singlets *via* RISC,^7^ which is in agreement with the quantum chemistry calculations and previous reports devoted to similar dyes.

Looking at the EL spectra in Fig. 6b, it can be seen that only the emission from the quasi-equatorial conformer is observed. Previous reports have provided evidence of the strong influence of the OLED architecture on the EL spectra due to optical cavity effects.^48–53^ In order to examine the potential impact of these optical cavity effects on the dual emission spectrum of (T-TRZ)-PTZ, we carried out optical simulations to see if these optical cavity effects could hide the emission band at 427 nm. These optical simulations were performed using SETFOS 4.2 software in the wavelength range between 380 and 750 nm. The light-emitting process taking place in the emissive layer of the OLED is simulated based on the model of emissive dipoles embedded within a multilayer stack.^54^ Since the thicknesses of these layers are comparable to the wavelength of the emitted light, internal reflections at the different interfaces occur, leading to optical interference effects. To model these interference effects in the OLED, a transfer matrix method is used,^55^ taking into account the optical indices of the emissive layer determined by variable angle spectroscopic ellipsometry.^56^ It should be noted that the calculation was carried out by assuming randomly distributed transition dipole moment approximated by a Dirac distribution placed at the interface between the emissive layer and the mCBP one.

**Fig. 7:**
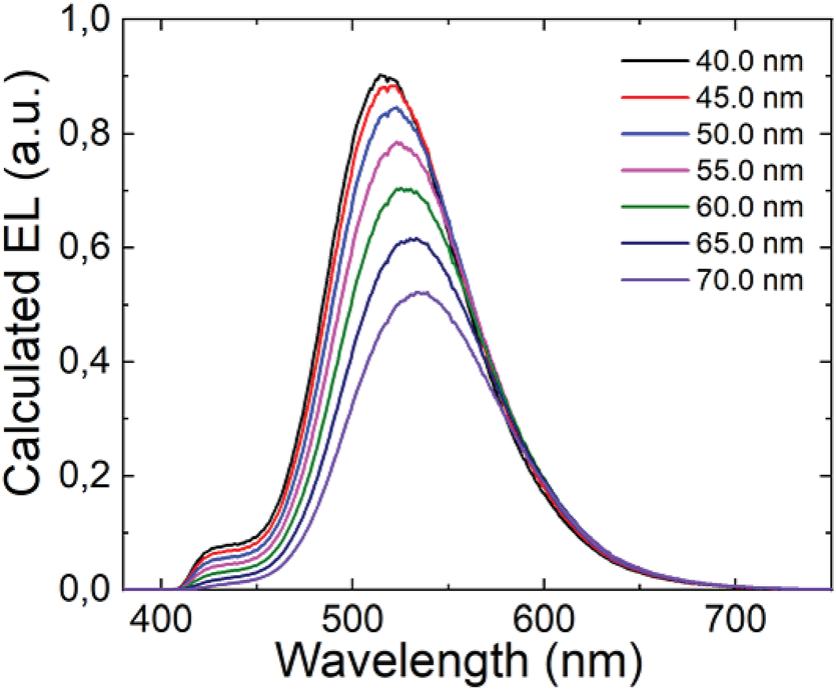
Calculated EL spectra of the (T-TRZ)-PTZ OLED with different thicknesses of TPBi.

It is well established that the distance between the emitting zone and the metal cathode predominantly affects the interference conditions at the position of the emissive zone. In this context, we decided to vary the thickness of the TPBi layer in the (T-TRZ)-PTZ OLED and see how this affects the intensity of the band at 427 nm in the EL spectrum. Fig. 7 shows the calculated EL spectrum of the OLED for different thicknesses of the TPBi film (varying from 40 to 70 nm). The results demonstrate that the EL spectrum is red-shifted and the intensity of the band at 427 nm is decreased when the TPBi thickness increases. In other words, the fact that the emission peak at 427 nm was not observed in the EL spectrum can be explained by these interference effects. This clearly demonstrates that the architecture of the OLED should be carefully designed to optimize the dual emission in the EL spectrum.

**Fig. 8:**
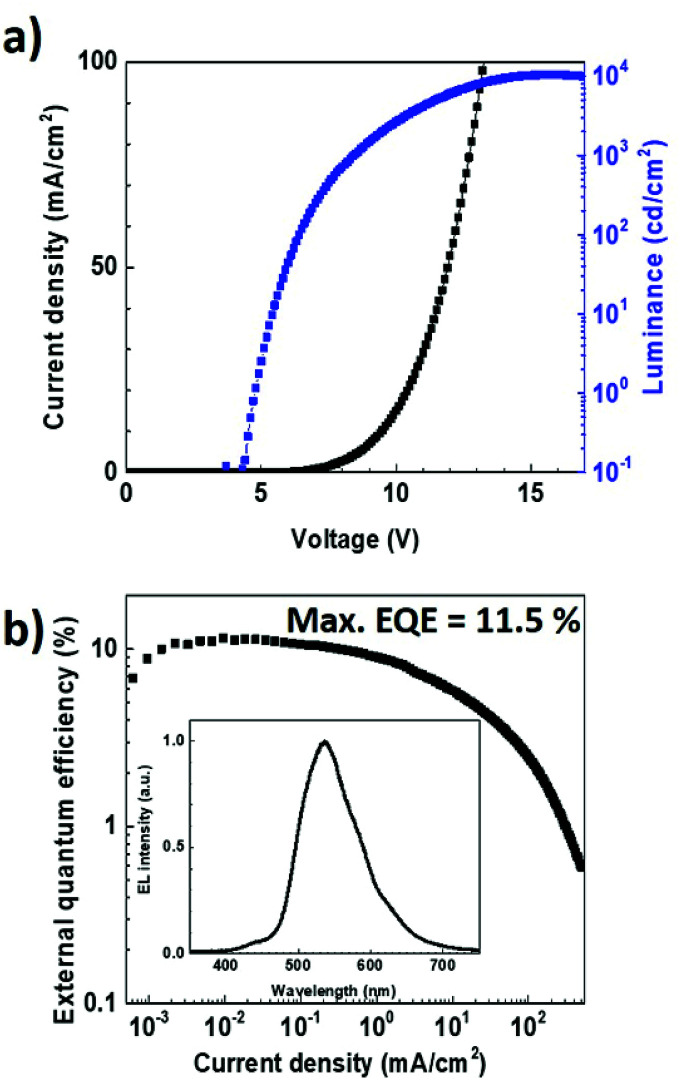
(a) Current density–voltage-luminance characteristic and (b) external quantum efficiency as a function of the current density for the OLED with a TPBi film thickness of 35 nm. The electroluminescence spectrum obtained for a current density of 100 mA cm^−2^ in shown in the inset.

To verify experimentally these modelling results, we fabricated an OLED using the same multilayer structure but with a thickness of 35 nm for the TPBi layer. The electroluminescence data obtained with such a device architecture are reported in Fig. 8. Noticeably, in contrast to what was achieved with a TPBi film thickness of 55 nm, the emission peak at 427 nm can be observed in the EL spectrum. This fully supports the results obtained from the optical simulations and demonstrates the importance of the optical cavity effects on the dual emission from TADF OLEDs. The *J*–*V* curve and the EQE of the TADF OLED were also measured as a function of the current density. The maximum EQE value and luminance measured for this OLED were found to be as high as 11.5% and 10 370 cd cm^−2^, respectively, providing evidence that not only the dual emission but also the overall performances of the EL device was improved after the optimization of the TPBi film thickness.

## Conclusions

In summary, we designed and synthesized the novel TADF molecule (T-TRZ)-PTZ, which contains 4,6-bis-phenyl phenothiazine as donor units and 2-thiophene-1,3,5-triazine as acceptor unit. A simple and low-cost synthetic method based on cobalt catalysed cross-coupling was used to produce this light-emitting compound. This method fits with the asset of reducing OLED cost thanks to the used low-cost reagents. The photoluminescence spectrum of (T-TRZ)-PTZ in solution and film exhibits a dual emission which is due to the incorporation of phenothiazine unit in the molecular structure, resulting in the existence of two stable molecular conformations for (T-TRZ)-PTZ. The electroluminescence properties of (T-TRZ)-PTZ are then characterized in OLEDs and the best device exhibited maximum external quantum efficiency and luminance values of 11.5% and 10 370 cd m^−2^, respectively. Based on experimental and optical simulations results, this study also provides new useful evidences that the OLED architecture must be carefully designed to optimize the dual emission in the electroluminescence spectrum. This latter result should be extremely relevant to further studies devoted to the development of high-efficiency white OLEDs based on single-component organic dual light-emitting molecules.

## Experimental section

### General information

All chemicals are reagent/analytical grade and used without further purification. Chromatographic purification of the products was accomplished using forced-flow chromatography on Geduran Silica Gel 60 40–63 mesh according to the method of Still. Thin-layer chromatography (TLC) was performed on Merck 0.20 mm silica gel 60-F254 plates. Visualisation of the developed chromatogram was performed by using UV light (254 nm). ^1^H, ^13^C, ^19^F NMR spectra were recorded on Bruker AC-300 (300 MHz, 75 MHz and 282 MHz respectively) as noted, and are internally referenced to residual solvent signals (CDCl_3_). Data for ^1^H, ^13^C and ^19^F NMR are reported as follows: chemical shift (*δ* ppm), multiplicity (s = singlet, d = doublet, t = triplet, q = quadruplet, m = multiplet), coupling constant and integration. High resolution mass spectra were obtained from the Ecole Polytechnique; Mass Spectral facility. Melting points were determined with a Stuart Automatic Melting Point SMP 40 and are uncorrected. All of the starting materials were purchased from the usual suppliers (Sigma-Aldrich, Alfa Aesar, and Acros Organics).

### Synthesis

#### 2,4-Dichloro-6-(thiophen-2-yl)-1,3,5-triazine

CoBr_2_ (437 mg, 2 mmol, 0.1 equiv.) and Zn dust (3.9 g, 60 mmol, 3 equiv.) were added in MeCN (16 mL). The mixture was stirred at room temperature for 1 hour and 2-bromothiophene (1.92 mL, 20 mmol, 1 equiv.) was added and the reaction was stirred at room temperature for 6 hours. The reaction was stirred to give the thiophen-2-ylzinc(ii) bromide reagent (100% in GC). 2,4,6-Trichloro-1,3,5-triazine (4.43 g, 24 mmol, 1.2 equiv.) was added. The mixture was stirred for 18 h at room temperature. The reaction was quenched by addition of saturated aqueous NH_4_Cl solution (80 mL) followed by extraction with dichloromethane (4 × 100 mL). The combined organic layers were dried over MgSO_4_ and after filtration the solvents were evaporated in vacuum. Recrystallization from methanol and ethyl acetate afforded the product in 54% (2.4 g) yield as a yellow solid.

^1^H-NMR (300 MHz, CDCl_3_): *δ*/ppm = 8.27 (dd, *J* = 3.9, 1.2 Hz, 1H), 7.77 (dd, *J* = 5.0, 1.2 Hz, 1H), 7.25–7.21 (m, 1H). ^13^C-NMR (75 MHz, CDCl_3_): *δ*/ppm = 171.8, 170.3, 138.1, 136.3, 135.2, 129.4. HRMS (EI+) (C_7_H_3_Cl_2_N_3_S): calculated *m*/*z*: 230.9425, found: 230.5421.

#### 2,4-Bis(4-fluorophenyl)-6-(thiophen-2-yl)-1,3,5-triazine

CoBr_2_ (109 mg, 0.5 mmol, 0.1 equiv.) and zinc dust (1.08 g, 16.5 mmol, 3.3 equiv.) were added in acetonitrile (4 mL). The mixture was stirred at room temperature for 1 h and 4-bromofluorobenzene (0.55 mL, 5 mmol, 1 equiv.) was added and the reaction was stirred at room temperature for 1 hour to give the (4-fluorophenyl)zinc bromide reagent (80% in GC). 2,4-Dichloro-6-(thiophen-2-yl)-1,3,5-triazine was added (274 mg, 1.19 mmol, 0.35 equiv.). The mixture was stirred for 18 h at room temperature. The reaction was quenched by addition of saturated aqueous NH_4_Cl solution (40 mL) followed by extraction with dichloromethane (4 × 40 mL). The combined organic layers were dried over MgSO_4_ and after filtration the solvents were evaporated in vacuum. Recrystallization from methanol and ethyl acetate afforded the product in 60% (210 mg) yield as a white solid.

^1^H-NMR (300 MHz, CDCl_3_): *δ*/ppm = 8.73 (dd, *J* = 9.0, 5.6 Hz, 4H), 8.35 (dd, *J* = 3.8, 1.2 Hz, 1H), 7.66 (dd, *J* = 5.0, 1.2 Hz, 1H), 7.27–7.20 (m, 5H). ^13^C-NMR (75 MHz, CDCl_3_): *δ*/ppm = 170.7, 168.2, 166.0 (d, *J* = 253.2 Hz), 142.0, 132.5, 132.1 (d, *J* = 2.9 Hz), 131.8, 131.4 (d, *J* = 9.1 Hz), 128.7, 115.9 (d, *J* = 21.8 Hz). ^19^F-NMR (282 MHz, CDCl_3_): *δ*/ppm = −106.81. HRMS (EI+) (C_19_H_11_F_2_N_3_S): calculated *m*/*z*: 351.0642, found: 351.0641.

#### 10,10′-((6-(Thiophen-2-yl)-1,3,5-triazine-2,4-diyl)bis(4,1-phenylene))bis(10*H*-phenothiazine)

To a solution of phenothiazine (52 mg, 0.26 mmol, 2.2 equiv.) in dry DMF under nitrogen was added sodium hydride (20 mg, 60% dispersion in mineral oil, 0.53 mmol, 4.4 equiv.). The reaction mixture is stirred at room temperature for 2 hours, a solution of 2,4-bis(4-fluorophenyl)-6-(thiophen-2-yl)-1,3,5-triazine (41 mg, 0.12 mmol, 1 equiv.) in 1 mL of DMF was added and the mixture is stirred at 100 °C for 18 hours. The reaction was quenched by addition of water (10 mL) followed by extraction with ethyl acetate (3 × 10 mL). The combined organic layers were washed with a 1 M aqueous lithium chloride solution (3 × 20 mL), then with a 2 M aqueous NaOH solution (3 × 20 mL), dried over MgSO_4_ and after filtration the solvents were evaporated in vacuum. Recrystallization from ethyl acetate and petroleum ether afforded the product in 54% (46 mg) yield as a yellow solid.

^1^H-NMR (300 MHz, CDCl_3_): *δ*/ppm = 8.79 (d, *J* = 8.6 Hz, 4H), 8.36 (d, *J* = 3.7 Hz, 1H), 7.65 (dd, *J* = 5.0, 1.2 Hz, 1H), 7.44 (d, *J* = 8.6 Hz, 4H), 7.26–7.24 (m, 1H), 7.21 (dd, *J* = 7.5, 1.6 Hz, 4H), 7.06 (td, *J* = 7.5, 1.6 Hz, 4H), 6.98 (td, *J* = 7.5, 1.2 Hz, 4H), 6.74 (dd, *J* = 7.5, 10.2 Hz, 4H). ^13^C-NMR (75 MHz, CDCl_3_): *δ*/ppm = 170.9, 168.1, 146.8, 143.1, 142.2, 133.1, 132.4, 131.6, 131.2, 128.7, 127.7, 127.2, 125.7, 125.2, 124.1, 120.3. HRMS (EI+) (C_43_H_27_N_5_S_3_): calculated *m*/*z*: 709.1429, found: 709.1416.

### Photophysical measurements

For the photophysical measurements, the degassed toluene solution of (T-TRZ)-PTZ was placed in a quartz cuvette. In parallel, the organic thin film was deposited by thermal evaporation onto a precleaned fused silica substrate. UV-visible absorption and steady-state PL spectra were measured using a UV-2550 spectrometer (Shimadzu) and a Fluoromax-4 spectrophotometer (Horiba Scientific), respectively. The transient PL decay was measured by a streak camera (Hamamatsu Photonics C4334). The samples were placed under vacuum (<4 × 10^−1^ Pa) and photoexcited by a nitrogen gas laser (Ken-X, Usho Optical Systems) delivering 500 ps pulses at a repetition rate of 20 Hz and emitting at 337 nm.

### OLED fabrication and measurements

The devices were prepared using precleaned indium tin oxide (ITO) substrates. The different organic layers were sequentially thermally evaporated under vacuum lower than 3 × 10^−4^ Pa. Finally, the LiF/Al electrodes were deposited through a shadow mask on top of the multilayer structure. The device active area was 4 mm^2^. To avoid any degradation and emission quenching due to oxygen and moisture, the OLEDs were encapsulated in a glovebox filled with nitrogen. The *J*–*V*–*L* characteristics were collected using a source meter (Keithley 2400, Keithley Instruments Inc.) and an absolute external quantum efficiency measurement system (C9920-12, Hamamatsu Photonics). An optical fiber connected to a spectrometer (PMA-12, Hamamatsu Photonics) was used to record the electroluminescence spectra.

## Conflicts of interest

There are no conflicts to declare.

## Supplementary Material

RA-009-C8RA10393C-s001
