# Supplementary material for: Control of the dual emission from a thermally activated delayed fluorescence emitter containing phenothiazine units in organic light-emitting diodes
Source: RSC Adv. 2019 Feb 4;9(8):4336–43. doi: 10.1039/c8ra10393c (PMC9060425; doi:10.1039/c8ra10393c)
Supplement: RA-009-C8RA10393C-s001 [file RA-009-C8RA10393C-s001.pdf]

Electronic Supplementary Material (ESI) for RSC Advances.
This journal is © The Royal Society of Chemistry 2019

Control of the dual emission from a thermally activated delayed fluorescence emitter containing phenothiazine units in organic light-emitting diodes

Ikbal Marghad[a,b], Fatima Bencheik [a,c], Chao Wang,[d] Sophia Manolikakes [b], Alice Rérat [b], Corinne Gosmini[b], Dae hyeon Kim [a,c], Jean-Charles Ribierre [a,c], and Chihaya Adachi [a,c]

[a] *Center for Organic Photonics and Electronics Research (OPERA), Kyushu University, Motooka 744, Nishi, Fukuoka 819-039, Japan*

[b] *Laboratoire de chimie moléculaire LCM Ecole Polytechnique, CNRS, 91128 Palaiseau Cedex, France*

[c] *Japan Science and Technology Agency (JST), ERATO, Adachi Molecular Exciton Engineering Project, Fukuoka 819-0395, Japan*

[d] *Department of Polymer Science and Engineering, Zhejiang University, Hangzhou 310027, China*

Contents

S1: ^{1}H-NMR, ^{13}C-NMR of the 2,4-dichloro-6-(thiophen-2-yl)-1,3,5-triazine.

S2:^{1}H-NMR, ^{13}C-NMR ,^{19}F-NMR of the 2,4-bis(4-fluorophenyl)-6-(thiophen-2-yl)-1,3,5-triazine.

S3: ^{1}H-NMR, ^{13}C-NMR of the 10,10'-((6-(thiophen-2-yl)-1,3,5-triazine-2,4-diyl)bis(4,1-phenylene))bis(10H-phenothiazine) or (T-TRZ)-PTZ.

Table S1: Cartesian coordinates of conformer A and E of (T-TRZ)-PTZ optimized at the B3LYP/6-31G(d) level.

Table S2: The comparison of total energy (E) of conformer A and E and their energy difference (ΔE) calculated at the B3LYP/6-31G(d) level.

Table S3: Configuration interactions of S_1 and T_1 transitions for conformer A and E calculated at the B3LYP/6-31G(d) level.

S1: ^{1}H-NMR, ^{13}C-NMR of the 2,4-dichloro-6-(thiophen-2-yl)-1,3,5-triazine

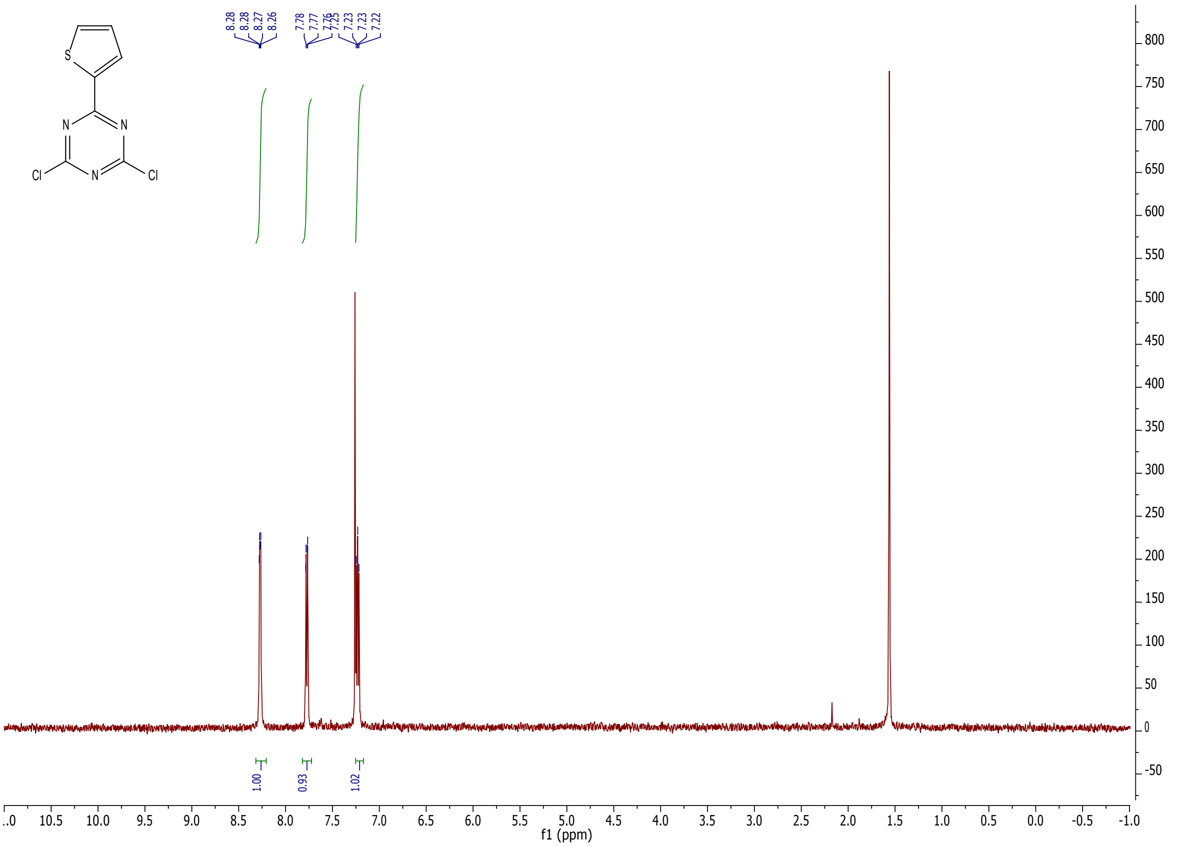

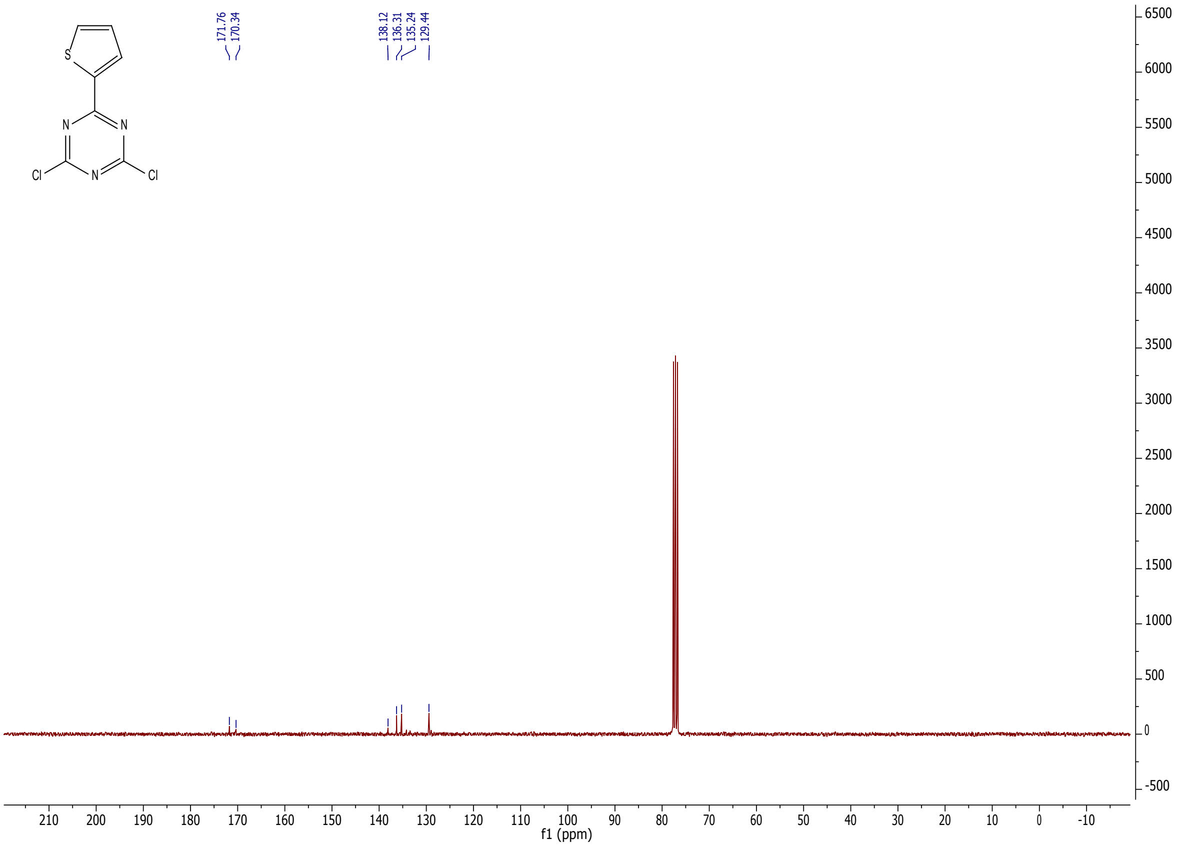

S2:1H-NMR, ^{13}C-NMR ,^{19}F-NMR of the 2,4-bis(4-fluorophenyl)-6-(thiophen-2-yl)-1,3,5-triazine

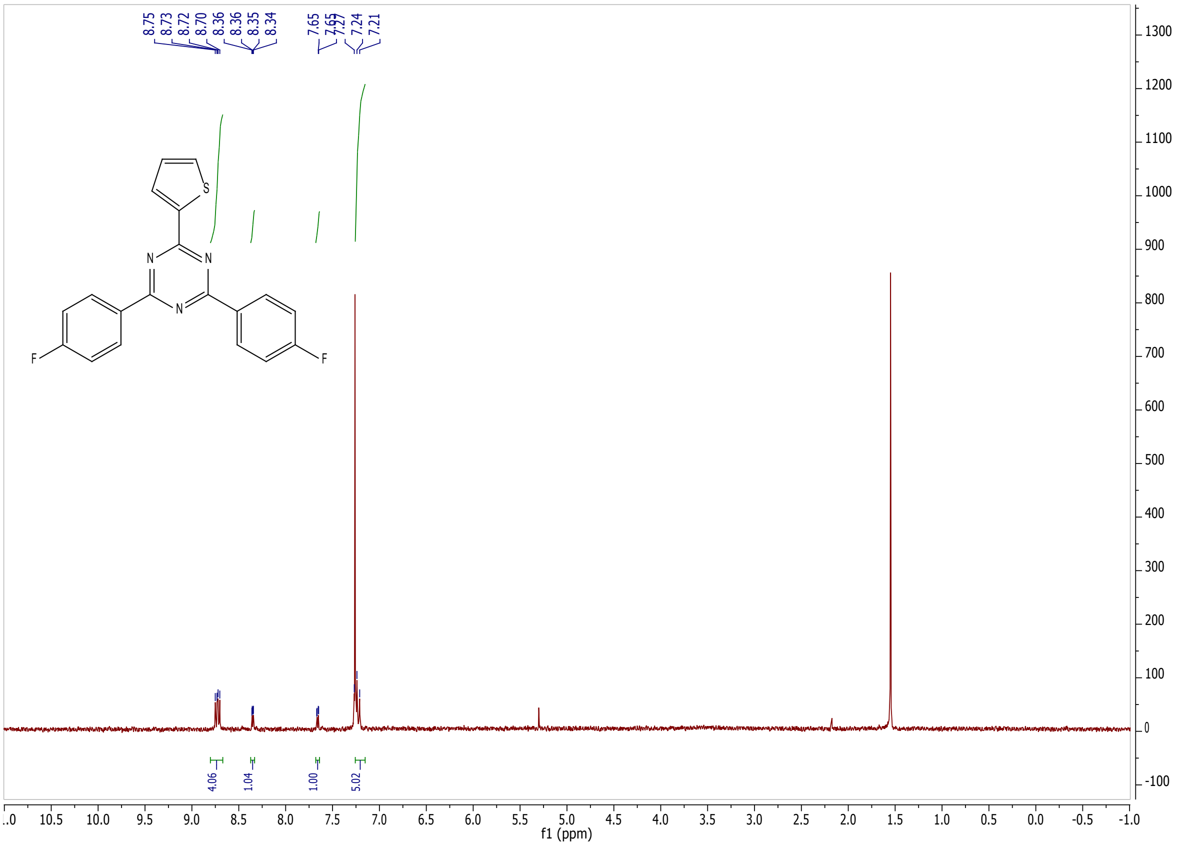

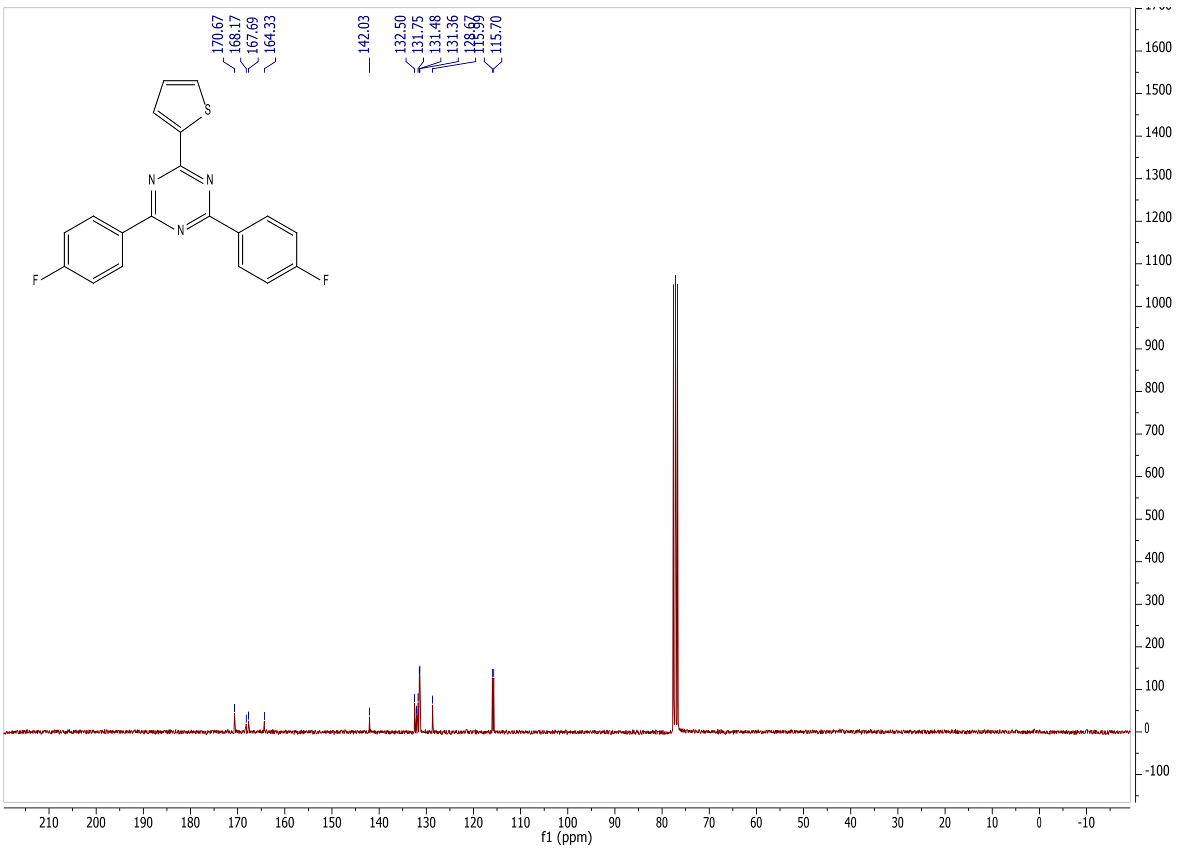

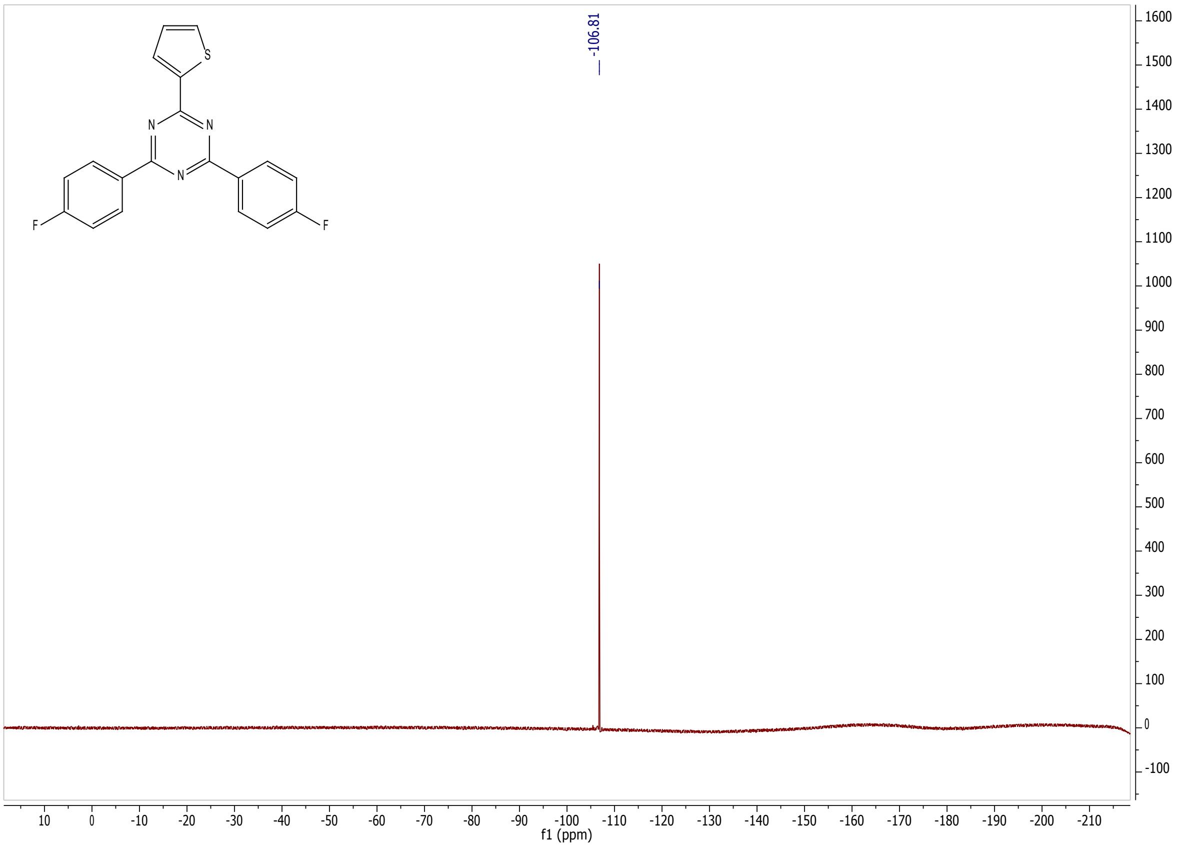

-106.81
N
N
N
S
F
F
f1 (ppm)

S3: ^{1}H-NMR, ^{13}C-NMR of the 10,10'-((6-(thiophen-2-yl)-1,3,5-triazine-2,4-diyl)bis(4,1-phenylene))bis(10H-phenothiazine)

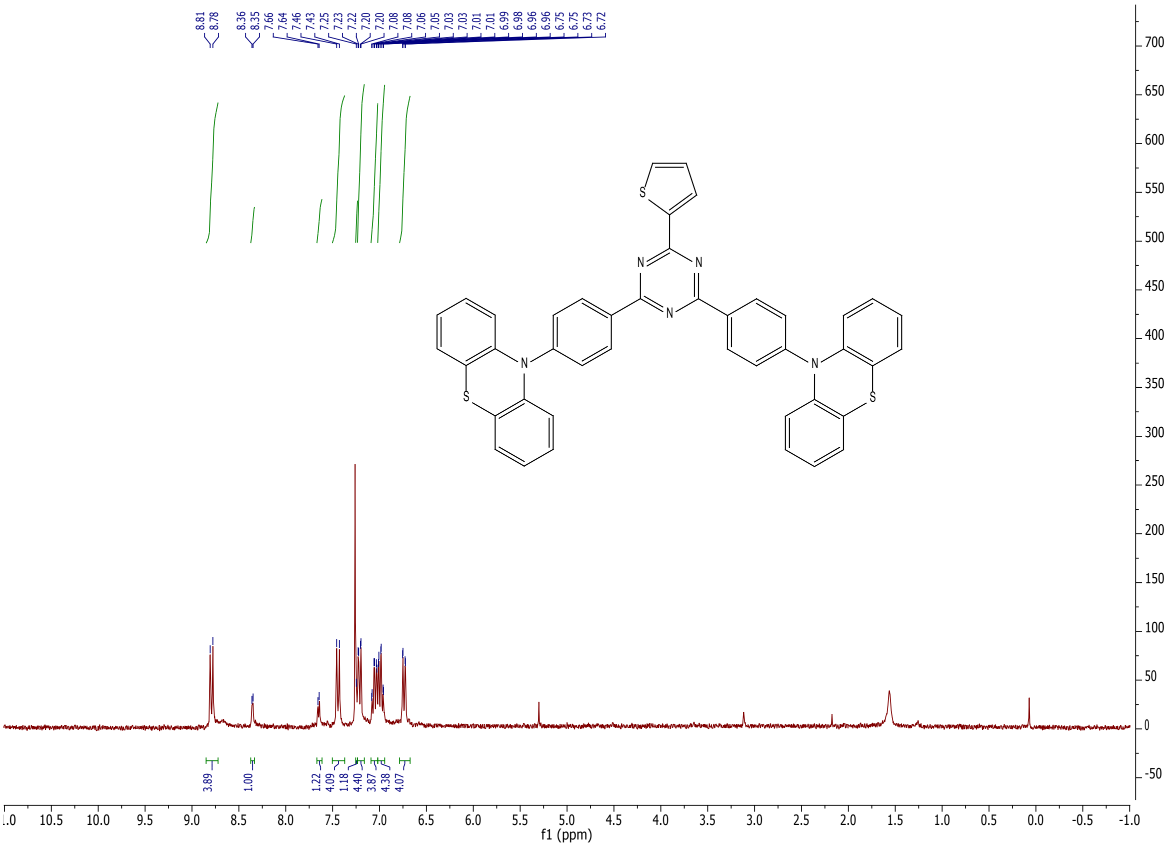

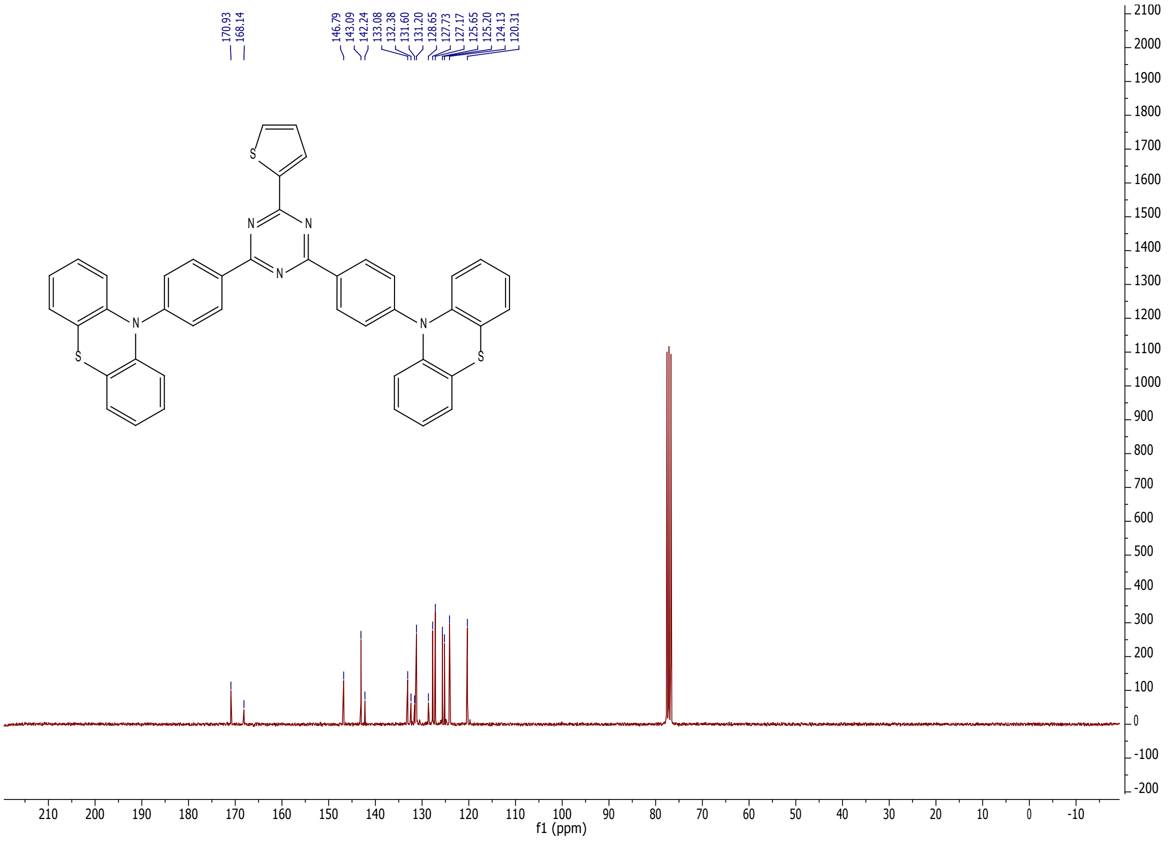

Table S1: Cartesian coordinates of conformer A and E of (T-TRZ)-PTZ optimized at the B3LYP/6-31G(d) level.

Conformer A

Atomic Symbol	Cartesian Coordinates (Å)		
	X	Y	Z
C	1.113056	1.700702	-0.076587
C	-0.005198	3.675034	0.008575
C	-1.161695	1.726612	0.089118
N	-1.200055	3.071608	0.094376
N	1.175525	3.045099	-0.078268
N	-0.032005	1.002925	0.005266
C	-2.441899	1.002405	0.181710
C	-3.661369	1.691253	0.265964
C	-2.486696	-0.399884	0.178553
C	-4.869599	1.016736	0.362924
H	-3.651259	2.775409	0.249012
C	-3.687967	-1.086767	0.275006
H	-1.557705	-0.952494	0.093256
C	-4.911991	-0.393528	0.386616
H	-5.786295	1.590651	0.404435
H	-3.674707	-2.168661	0.247309
C	2.378519	0.952993	-0.170733
C	3.610946	1.619465	-0.250338
C	2.395996	-0.449948	-0.173762
C	4.805817	0.921863	-0.348940
H	3.622456	2.703576	-0.228493
C	3.583836	-1.159273	-0.272214
H	1.456406	-0.984933	-0.091815
C	4.821052	-0.488854	-0.379457

H	5.733324	1.478327	-0.386643
H	3.549935	-2.240825	-0.249518
C	0.013539	5.135116	0.009631
C	-1.055791	5.999115	0.086283
S	1.523552	6.008057	-0.095453
C	-0.668584	7.365768	0.061073
H	-2.076415	5.643562	0.157391
C	0.691239	7.523450	-0.034441
H	-1.366454	8.194553	0.111394
H	1.250650	8.449232	-0.072259
C	7.303922	-0.553663	-0.425827
C	6.084416	-2.627271	-0.357879
C	8.180235	-0.872725	0.623877
C	7.718976	0.346646	-1.414025
C	6.819734	-3.186962	0.699381
C	5.471130	-3.474121	-1.288583
C	9.432673	-0.257612	0.709114
C	8.957745	0.978321	-1.313295
H	7.056845	0.555676	-2.248399
C	6.894369	-4.574822	0.849733
C	5.525183	-4.857273	-1.122317
H	4.942144	-3.037509	-2.129946
C	9.810856	0.683935	-0.246893
H	10.106272	-0.524186	1.518329
H	9.261446	1.689472	-2.076020
H	7.478412	-4.994656	1.663534
C	6.228494	-5.407891	-0.048106
H	5.030016	-5.504245	-1.840650
H	10.780431	1.167874	-0.172131
H	6.280157	-6.485621	0.077298

C	-7.395484	-0.410313	0.436080
C	-6.216834	-2.506795	0.352139
C	-8.277926	-0.703981	-0.615924
C	-7.792627	0.490468	1.431187
C	-6.962826	-3.043825	-0.709357
C	-5.620283	-3.372563	1.276258
C	-9.517960	-0.063554	-0.696248
C	-9.018708	1.147214	1.335559
H	-7.126531	0.679622	2.267180
C	-7.064510	-4.428748	-0.870480
C	-5.701266	-4.753079	1.099283
H	-5.083099	-2.952861	2.121036
C	-9.877464	0.877905	0.267035
H	-10.196771	-0.310591	-1.507306
H	-9.308521	1.858095	2.103936
H	-7.656564	-4.830837	-1.687443
C	-6.415050	-5.281530	0.020859
H	-5.218876	-5.415135	1.812539
H	-10.837393	1.381287	0.196249
H	-6.487677	-6.357041	-0.112886
N	6.032765	-1.204799	-0.489511
N	-6.137355	-1.086617	0.494725
S	-7.796319	-1.926335	-1.826365
S	7.674492	-2.094556	1.825016

Conformer E

Atomic Symbol	Cartesian Coordinates (Å)		
	X	Y	Z
C	1.112006	1.673736	0.000114
C	-0.006835	3.649527	0.000101

C	-1.162771	1.698986	0.000138
N	-1.203798	3.038814	0.000120
N	1.176172	3.012968	0.000105
N	-0.032711	0.973272	0.000135
C	-2.454478	0.968841	0.000123
C	-3.667975	1.674440	0.000225
C	-2.480608	-0.433759	-0.000001
C	-4.880310	0.991239	0.000185
H	-3.644844	2.758085	0.000314
C	-3.693719	-1.116762	-0.000036
H	-1.542840	-0.976992	-0.000072
C	-4.900227	-0.410329	0.000049
H	-5.818902	1.537792	0.000263
H	-3.720428	-2.201951	-0.000138
C	2.389671	0.920812	0.000077
C	3.615451	1.605208	0.000152
C	2.390048	-0.482053	-0.000028
C	4.815053	0.899746	0.000096
H	3.612704	2.689184	0.000243
C	3.590543	-1.186777	-0.000054
H	1.442544	-1.008203	-0.000086
C	4.809551	-0.501928	-0.000001
H	5.763290	1.429347	0.000123
H	3.597841	-2.272265	-0.000118
C	0.011409	5.105096	0.000124
C	-1.063883	5.967575	0.000197
S	1.523618	5.980923	0.000089
C	-0.678338	7.333337	0.000201
H	-2.087026	5.612175	0.000227
C	0.685467	7.492437	0.000192

H	-1.378488	8.161360	0.000245
H	1.244583	8.419145	0.000208
C	6.697505	-1.466600	-1.238241
C	6.697579	-1.466662	1.238096
C	7.679470	-2.470017	-1.354317
C	6.398890	-0.700189	-2.375580
C	7.679572	-2.470069	1.354041
C	6.399029	-0.700335	2.375506
C	8.363711	-2.661033	-2.555395
C	7.057667	-0.926280	-3.584643
H	5.639334	0.070080	-2.321328
C	8.363909	-2.661146	2.555053
C	7.057904	-0.926487	3.584506
H	5.639450	0.069920	2.321373
C	8.049461	-1.899459	-3.681118
H	9.130885	-3.428676	-2.606260
H	6.794185	-0.323568	-4.449367
H	9.131100	-3.428779	2.605812
C	8.049730	-1.899646	3.680848
H	6.794467	-0.323835	4.449286
H	8.572683	-2.069362	-4.617362
H	8.573028	-2.069590	4.617041
C	-6.805495	-1.340081	-1.238241
C	-6.805373	-1.340470	1.238223
C	-7.806638	-2.324373	-1.354347
C	-6.491884	-0.579929	-2.375756
C	-7.806503	-2.324795	1.354125
C	-6.491651	-0.580666	2.375938
C	-8.494277	-2.502311	-2.555482
C	-7.154733	-0.793439	-3.584892

H	-5.717297	0.175231	-2.321638
C	-8.494015	-2.503119	2.555272
C	-7.154372	-0.794560	3.585076
H	-5.717074	0.174516	2.321974
C	-8.165236	-1.747158	-3.681314
H	-9.276071	-3.255053	-2.606353
H	-6.879325	-0.196304	-4.449773
H	-9.275798	-3.255883	2.605989
C	-8.164859	-1.748315	3.681306
H	-6.878881	-0.197691	4.450114
H	-8.691486	-1.907136	-4.617602
H	-8.691010	-1.908588	4.617600
N	6.030470	-1.260406	-0.000050
N	-6.134744	-1.146414	-0.000013
S	-8.119535	-3.437775	-0.000290
S	7.970854	-3.589023	-0.000169

Table S2: The comparison of total energy (E) of conformer A and E and their energy difference (ΔE) calculated at the B3LYP/6-31G(d) level.

Conformer	E (au)	ΔE (eV)
A	-3123.18856882	-0.038
E	-3123.18716211	

Table S3: Configuration interactions of S_1 and T_1 transitions for conformer A and E calculated at the B3LYP/6-31G(d) level.

Conformer	Excited state	Initial → Final	Contribution
E	S_1	HOMO → LUMO	19 %
		HOMO → LUMO+1	79 %
	T_1	HOMO → LUMO	19 %
		HOMO → LUMO+1	78 %
A	S_1	HOMO → LUMO	14 %
		HOMO → LUMO+1	79 %
	T_1	HOMO → LUMO	23 %
		HOMO-1 → LUMO+1	45 %
